# Quality of life in patients with spinal muscular atrophy in Brazil: patient self-assessment and carer perception

**DOI:** 10.1590/1984-0462/2025/43/2024073

**Published:** 2025-01-17

**Authors:** Maíra Coelho, Marise Bueno Zonta, Salmo Raskin, Silvia Valderramas

**Affiliations:** a Universidade Federal do Paraná, Curitiba, Paraná, Brazil.; b Laboratório de Genética, Curitiba, Paraná, Brazil.

**Keywords:** Child neurology, Family impact, Muscular atrophy spinal, Patient-reported outcomes, Neurologia infantil, Impacto familiar, Atrofia muscular espinhal, Resultados relatados pelo paciente

## Abstract

**Objectives:**

The aim of this study was to assess the perception of quality of life of patients with spinal muscular atrophy (SMA) and investigate whether there is a correlation between patients’ perception and that of their carers.

**Methods:**

Cross-sectional analytical observational study. In the first part, socioeconomic, demographic, clinical, and treatment information were collected from patients diagnosed with SMA, regardless of type, sex, or age. SMA type 1 does not sit; SMA type 2 sits; SMA type 3 walks; and SMA type 4 begins in adulthood. In the second part of the study, patients aged between 2 and 25 years and their caregivers responded to the Pediatric Quality of Life Inventory 4.0 questionnaire.

**Results:**

Of the 235 families recruited, 167 were eligible to respond to the questionnaire, 115 caregivers and 49 patients were included. The results point to a different perception of quality of life between patients and caregivers. Patients with SMA type 2 perceive more impaired physical and emotional capacity compared to SMA type 3 and 1, respectively. As for caregivers, the perception of quality of life in relation to physical and social capacity and the total score are worse for patients with SMA type 1 compared to other types. Perceptions correlate with emotional capacity in SMA type 2 and the total aspect in SMA type 3.

**Conclusions:**

Patients with SMA type 2 had a worse perception of their quality of life than other patients. Perception differed between patients and their caregivers, with the former having a better perception than the latter.

## RESUMO

**Objetivo:**

Avaliar a percepção da qualidade de vida dos pacientes com atrofia muscular espinhal (AME) e investigar se existe correlação entre a percepção dos pacientes e a de seus cuidadores.

**Métodos:**

Estudo observacional analítico transversal. Na primeira parte foram coletadas informações socioeconômicas, demográficas, clínicas e de tratamento de pacientes com diagnóstico de AME, independentemente do tipo, sexo ou idade. Paciente com AME tipo 1 não senta; AME tipo 2 senta; AME tipo 3 caminha; e AME tipo 4 começa na idade adulta. Na segunda parte do estudo, pacientes com idade entre 2 e 25 anos e seus cuidadores responderam ao questionário de Qualidade de Vida Pediátrico — PeDsQL™ 4.0.

**Resultados:**

Das 235 famílias recrutadas, 167 foram elegíveis para responder ao questionário, 115 cuidadores e 49 pacientes. Os resultados apontam para uma percepção diferente de qualidade de vida entre pacientes e cuidadores. Pacientes com AME tipo 2 percebem maior comprometimento da capacidade física e emocional em comparação com AME tipo 3 e 1, respectivamente. Quanto aos cuidadores, a percepção da qualidade de vida em relação à capacidade física e social e ao escore total é pior para os pacientes com AME tipo 1 em comparação aos demais tipos. As percepções correlacionam-se na capacidade emocional na AME tipo 2 e no aspecto total na AME tipo 3.

**Conclusões:**

Pacientes com AME tipo 2 tiveram pior percepção de qualidade de vida do que outros pacientes. A percepção diferiu entre pacientes e seus cuidadores, tendo os primeiros uma percepção melhor que os segundos.

## INTRODUCTION

Spinal muscular atrophy (SMA) is a severe neuromuscular disorder inherited in an autosomal recessive pattern caused by a defect on chromosome 5q13.2. In most patients, the defect results in a homozygous deletion of the *SMN1* (survival motor neuron 1) gene.^1,2^ The condition is classified into four clinical types (1, 2, 3, and 4) based on age of onset and symptom severity.^1,2^

Patients with type 1 SMA, the most common type and most severe form of the disorder, in which symptom onset occurs at up to 6 months of age, are not able to sit and require respiratory support for the first 2 years of life. Patients with type 2 SMA, with symptom onset between 6 and 18 months of age, are able to sit but not walk. Patients with type 3 SMA, with symptom onset after 18 months of age, learn how to walk but may lose this ability. Patients with type 4 SMA normally present with muscle weakness from the second decade of life onward.^3-5^ Because it is a severe, chronic disorder characterized by progressive muscular weakness, patients with SMA can experience a compromised quality of life (QoL). The patient's perception of his or her physical, psychological (including emotional and cognitive), and social dimensions may differ from those of the carer.^6^

Assessment of health-related quality of life (HRQoL) is essential to understanding the disorder's impact and the effectiveness of treatment, which is an important outcome in clinical trials.^3,6-9^ There exist no studies in Brazil on the prevalence or epidemiology of the disorder or the HRQoL of SMA patients, making a multidisciplinary approach to treat these patients and develop public policies specifically for this population difficult.

The aim of this study was to assess the perception of patients’ QoL and to investigate whether there is a correlation between patients’ perception and that of their carers.

## METHOD

This is a cross-sectional analytical observational study. The project was assessed and approved by the Committee for Ethics in Research in Humans, Health Sciences Sector, UFPR, under Certificate of Presentation for Ethical Appreciation (CAAE) registration no. 39003720.4.0000.0102. In the first part of the study, participants were invited to respond to the form created by the researchers and to collect demographic data and details about the patient's socioeconomic situation, clinical condition, complications, and treatment, which included all individuals, regardless of sex or age, who were diagnosed with SMA type 1, 2, 3, or 4 and their caregivers who signed the Free and Informed Consent Form. In the second part of the study, patients diagnosed with SMA, aged between 2 and 25 years, and their caregivers responded to the Pediatric Quality of Life Inventory 4.0 (PedsQL™ 4.0) questionnaire, and patients aged less than 2 years or greater than 25 years were excluded from this study.

To recruit participants, invitations to take part in the study were posted on the Association of Friends of Spinal Muscular Atrophy's (AAME) social networks. Individuals who accepted the invitation first received a link to access the form prepared by the researchers.

Afterward, children, adolescents, and adults aged between 2 and 25 years with a diagnosis of SMA and their caregivers received the link to access the questionnaire (PedsQLTM 4.0), which was guided by one of the researchers. In the case of children between 2 and 4 years, only the carers answered the questionnaires. For those aged between 5 and 25 years, both patients and carers filled out the questionnaires.

HRQoL was assessed with the PedsQL™ 4.0 with permission from the owner of the copyright, Dr. James Varni, Texas A&M University (College Station, Texas). Permission was granted to use the questionnaires targeted by age group (2–4 years; 5–7 years; 8–12 years; 13–18 years; and 18–25 years) in the validated Brazilian Portuguese version.

The PedsQL™ 4.0 questionnaires consist of 23 items divided into four multidimensional domains: physical functioning (eight items), emotional functioning (five items), social functioning (five items), and school functioning (five items).^9,10^ A psychosocial health summary score is calculated by adding the scores for the items answered in the emotional functioning, social functioning, and school functioning domains and dividing the result by the number of items answered.^9,10^ For each item, the patient or carer is asked to answer on a 5-point Likert scale (0=never; 1=almost never; 2=sometimes; 3=almost always; 4=always).^11^ In accordance with the PedsQL™ scaling and scoring, which is available at http://www.pedsql.org/PedsQL-Scoring.pdf, the items are transformed linearly on a scale from 0 to 100 (0=100, 1=75, 2=50, 3=25, and 4=0) so that higher scores indicate a better perception of QoL.

This study intended to reach a sample size that was representative of SMA in Brazil. As there exist no studies describing this prevalence, the authors determined the representative sample considering studies carried out in some countries that describe the prevalence between 5 and 11.9%.^12-17^

The sample size for the present study was calculated with the following formula:^18^


$$\text{N}={\text{Z}}^{2*}[\text{incidence}*(1\text{-incidence})]{\text{/D}}^{2},$$

where Z=1.96; D=0 0.05 (effect size); type I error (α)=0.05; type II error (β)=0.05; and the prevalence of SMA is assumed to be 11.9%, as shown by Verhaart et al.^17^ Based on this formula, the sample size for this study was estimated to be 162. To avoid possible losses during follow-up that could compromise the power of the study, the number of patients was increased by 20%, giving a total sample size of 195.

The Shapiro-Wilk test was used to test the variables for normality. The results for age and PedsQL™ scores are expressed as mean and standard deviation. Categorical variables are described in terms of absolute frequencies and percentages. The scores for the different SMA types were compared with the Kruskal–Wallis non-parametric test, and for multiple comparisons of the SMA types, Dunn's post hoc test was used with Bonferroni-adjusted p-values.

Spearman's correlation coefficients were used to determine the association between questionnaire scores for patients and parents/carers. The scale of magnitudes for effect statistics proposed by Batterham and Hopkins was used to interpret the correlation coefficients: <0.1=trivial; 0.10–0.29=small; 0.30–0.49=moderate; 0.50–0.69=large; 0.70–0.90=very large; >0.90 = nearly perfect.^19^ The significance level was set at p<0.05. The data were organized in an Excel® spreadsheet and analyzed with IBM SPSS Statistics version 28.0. Armonk, NY: IBM Corp.

## RESULTS

During the recruitment period (August 2020 to May 2022), 600 families were invited to participate through the AAME site. A total of 235 families (39%) answered the invitations and filled out the initial collection form. These consisted of 128 patients with type 1 SMA (54.5%), 74 with type 2 SMA (31.5%), 29 with type 3 SMA (12.3%), and 4 with type 4 SMA (1.7%). Patient age varied from 4 months to 59 years, and 119 (51%) were female.

**Figure 1:**
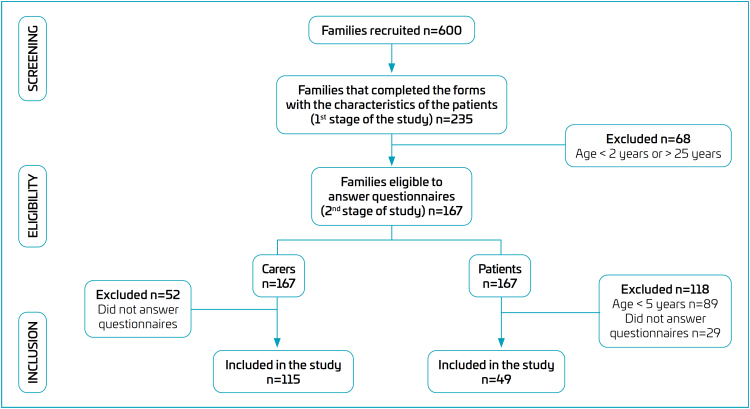
Flowchart of participant recruitment and assessment.

Of the 235 families, 167 (71%) were eligible to answer the PedsQL™ 4.0 questionnaires, and 68 (29%) were considered ineligible because the patients were younger than 2 years or older than 25 years. Of these 167 carers and 167 patients, 115 carers and 49 patients were included in the study (Figure 1).

The population sample (n=235) was a representative sample of all five regions in Brazil: Southeast (35.3%), South (31.5%), Northeast (18.3%), Midwest (8.5%), and North (6.4%).

Approximately one-third of the families (33.6%) live on just one minimum monthly wage, and in 44.5% the main carer was the mother. A total of 37.4% (n=88) only use the public health care system (SUS) and 31.5% (n=74) use health insurance. These and other socioeconomic characteristics are shown in Table 1.

**Table 1:** Socioeconomic characteristics of patients (n=235).

Variable			n (%)
Carer			
	Mother		100 (44.5)
	Father		4 (1.8)
	Mother and father		95 (42.2)
	Spouse		10 (4.4)
	Self-care		6 (2.7)
	Nursing team		5 (2.2)
	Other family members		5 (2.2)
Monthly income (x minimum monthly wage)			
	1		79 (33.6)
	2–3		59 (25.1)
	3–5		36 (15.3)
	5–10		38 (16.2)
	>10		23 (9.8)
Education			
	≤5 years (n=144)		
		Illiterate	144 (100)
	6–14 years (n=40)		
		Illiterate	10 (26.3)
		Did not finish primary school	27 (71.1)
		Finished primary school	1 (2.6)
	15–17 years (n=11)		
		Did not finish primary school	5 (45.5)
		Did not finish secondary school	5 (45.5)
		Did not finish university degree	1 (9)
	≥18 years (n=40)		
		Did not finish primary school	5 (12.5)
		Finished primary school	1 (2.5)
		Did not finish secondary school	3 (7.5)
		Finished secondary school	10 (25)
		Did not finish university degree	6 (15)
		Finished university degree	15 (37.5)
Access to health care			
	SUS		88 (37.4)
	Health insurance		74 (31.5)
	Private medical care		0 (0)
	SUS and private medical care		9 (3.8)
	Health insurance and private medical care		7 (3.0)
	Health insurance and private medical care and SUS		23 (9.8)
	SUS and health insurance		34 (14.5)

Data expressed as absolute (n) and relative (%) frequencies. SUS: Sistema Único de Saúde.

The demographic and clinical characteristics of the patients by SMA type are shown in Table 2.

**Table 2:** Demographic and clinical characteristics of patients by spinal muscular atrophy type (n=235).

Variable		Type 1 SMA (n=128)	Type 2 SMA (n=74)	Type 3 SMA (n=29)	Type 4 SMA (n=4)
Age (years)		3.9±3.7	13.6±12.3	20.1±16.6	41.3±11.9
Sex F, n (%)		61 (47.6)	41 (55.4)	18 (62.1)	2 (50.0)
Age range (years), n (%)					
	<2	39 (30.5)	1 (1.4)	0	0
	2–4	62 (48.5)	22 (29.7)	5 (17.2)	0
	5–7	12 (9.4)	14 (18.9)	6 (20.8)	0
	8–12	10 (7.8)	6 (8.1)	3 (10.3)	0
	13–18	4 (3.1)	9 (12.2)	3 (10.3)	0
	18–25	1 (0.7)	9 (12.2)	1 (3.5)	0
	>25	0	13 (17.5)	11 (37.9)	4 (100.0)
Use of specific medication					
	Do not use	19 (14.9)	32 (43.3)	18 (62.1)	4 (100.0)
	Spinraza	79 (61.7)	39 (52.7)	10 (34.5)	0
	Zolgensma	12 (9.4)	0	0	0
	Risdiplan	11 (8.6)	0	0	0
	Spinraza-Zolgensma	7 (5.4)	3 (4.0)	1 (3.4)	0
Use of equipment					
	None	9 (7.0)	33 (44.6)	20 (69)	2 (50.0)
	BiPAP	58 (45.3)	29 (39.2)	3 (10.4)	0
	Home ventilator	58 (45.3)	4 (5.4)	0	0
	CPAP	0	1 (1.4)	0	0
	Cough-Assist	78 (61.0)	14 (18.9)	0	0
	Ambu	75 (58.6)	24 (32.4)	8 (27.6)	2 (50.0)
	Oxygen	12 (9.4)	0	0	0
Physiotherapy					
	Motor	121 (94.5)	67 (90.6)	22 (75.9)	2 (50.0)
	Respiratory	115 (89.9)	40 (54.0)	7 (24.1)	0
Feeding route					
	Oral	29 (22.6)	71 (95.8)	29 (100.0)	4 (100.0)
	G-tube	76 (59.4)	1 (1.4)	0	0
	NG-tube	15 (11.7)	1 (1.4)	0	0
	Oral+G-tube	8 (6.3)	1 (1.4)	0	0
Need for hospitalization					
	No	80 (62.5)	56 (75.7)	28 (96.5)	3 (75.0)
	Yes	43 (33.6)	18 (24.3)	1 (3.5)	1 (25.0)
	Lives in the hospital	5 (3.9)	0	0	0
Cardiac arrest					
	Yes	31 (24.2)	8 (10.8)	1 (3.5)	0
	No	97 (75.8)	66 (89.2)	28 (96.5)	4 (100.0)

Data expressed as mean and standard deviation and absolute (n) and relative (%) frequencies. SMA: spinal muscular atrophy; F: female; BiPAP: bilevel positive airway pressure; CPAP: continuous positive airway pressure .

Table 3 shows the scores for each domain in the PeDsQL™ 4.0 by SMA type for patient self-assessment and carer assessment.

**Table 3:** PedsQL™ 4.0 – patient self-assessment (n=49) and carer assessment (n=115) by spinal muscular atrophy type.

PedsQL™ 4.0Domain	Type 1 SMA (n=10)	Type 2 SMA (n=25)	Type 3 SMA (n=14)	p-value*
Patient self-assessment (n=49) by SMA type				
Physical functioning^a^	38.8±27.3	29.0±16.9	48.2±22.4	0.026
Emotional functioning^b^	70.5±18.5	52.4±22.9	64.3±14.7	0.037
Social	55.0±25.3	68.0±17.7	58.9±24.0	0.229
School functioning	53.3±27.8	73.8±19.7	76.9±14.2	0.073
Psychosocial	61.2±20.7	64.7±13.7	66.8±13.4	0.610
Total score	53.4±21.3	52.3±11.6	60.4±14.6	0.149
Carer or parent assessment (n=115) by SMA type				
Physical functioning^a^,^b^,^c^	31.7±18.1	44.1±23.5	63.5±25.8	<0.001
Emotional functioning	68.3±15.8	65.4±19.2	61.6±21.3	0.724
Social functioning^d^	53.4±16.9	60.0±17.7	71.2±20.3	<0.001
School functioning	59±26.5	71.2±20.3	70.9±21.1	0.225
Psychosocial	61.1±14.1	64.5±12.8	67.2±15.7	0.115
Total score^e^,^f^	49.2±14.1	56.9±12.6	65.8±15.5	<0.001

SMA: spinal muscular atrophy. Results expressed as mean and standard deviation. For the analysis of the school functioning domain (patients) 9, 24, and 13 cases were included for types 1, 2, and 3 SMA, respectively, and for the analysis of the school functioning domain (career or parent), 23, 31, and 21 cases were included for types 1, 2, and 3 SMA, respectively.

* Non-parametric Kruskal-Wallis test and post hoc Dunn's test with p-values corrected by Bonferroni.

a Type 1 SMA vs. type 2 SMA (p=1); type 1 SMA vs. type 3 SMA (p=0.555); type 2 SMA vs. type 3 SMA (p=0.022)

b Type 1 SMA vs. type 2 SMA (p=0.050); type 1 SMA vs. type 3 SMA (p=1); type 2 SMA vs. type 3 SMA (p=0.202)

c Type 1 SMA vs. type 2 SMA (p=0.039); type 1 SMA vs. type 3 SMA (p<0.001); type 2 SMA vs. type 3 SMA (p=0.022)

d Type 1 SMA vs. type 2 SMA (p=0.104); type 1 SMA vs. type 3 SMA (p<0.001); type 2 SMA vs. type 3 SMA (p=0.139)

e Type 1 SMA vs. type 2 SMA (p=0.033); type 1 SMA vs. type 3 SMA (p<0.001); type 2 SMA vs. type 3 SMA (p=0.108)

The lowest and highest mean patient self-assessment scores for QoL were for physical functioning and school functioning, respectively. There were significant differences in the physical functioning and emotional functioning domains between SMA types. The results for these domains were therefore compared for two SMA types at a time. These comparisons revealed that the score for perceived emotional functioning was lower for type 2 SMA than for type 1 SMA, and that the score for perceived physical functioning was lower for type 2 SMA than for type 3 SMA.

As carers’ perceptions of physical and social functioning domains and total scores differed significantly between SMA types, pairwise comparisons were performed. The following statistically significant results were found: the score for perceived physical functioning was lower for type 1 SMA than for types 2 and 3 and lower for type 2 than for type 3. Similarly, the total score for type 1 SMA was lower than for types 2 and 3. Finally, the score for perceived social functioning for type 1 SMA was lower than for type 3.

**Figure 2:**
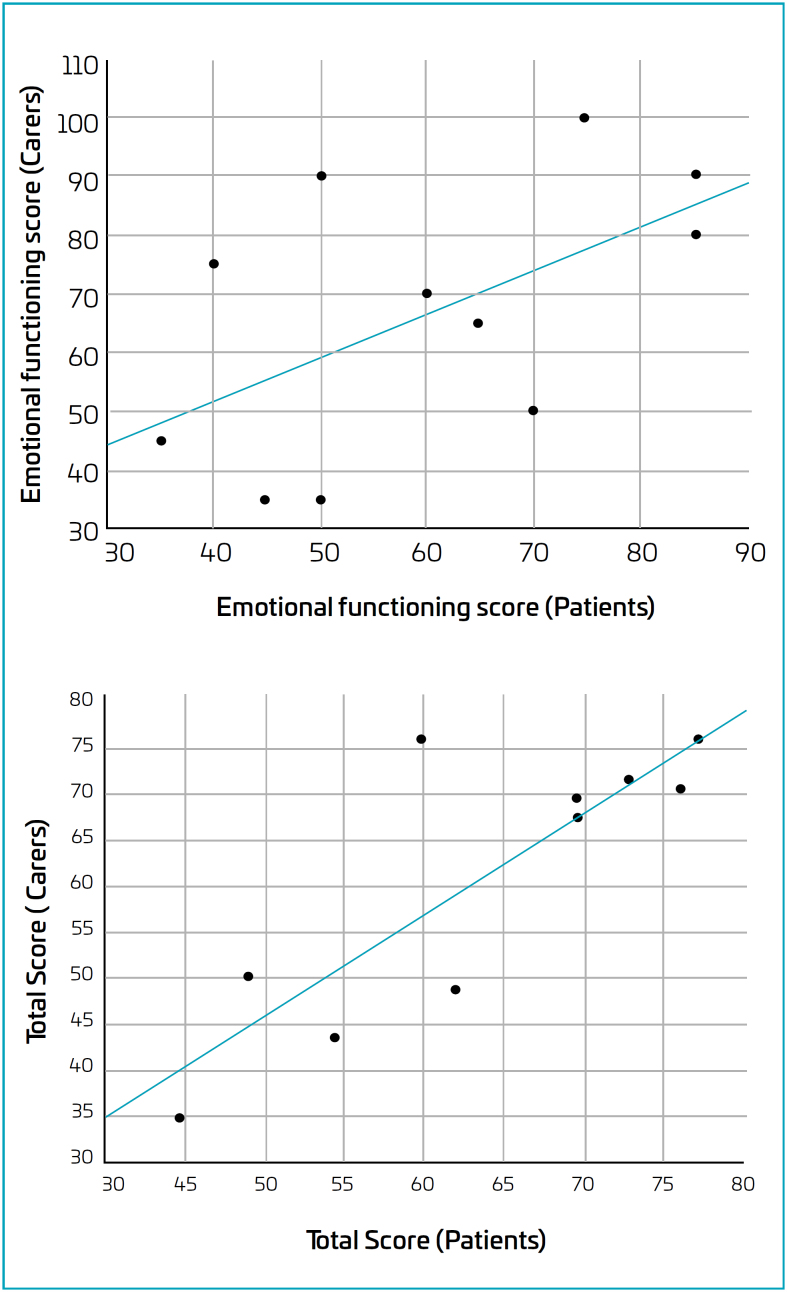
Scatter plots of carer and patient scores for the emotional functioning domain for type 2 and type 3 spinal muscular atrophy. Each point corresponds to a single case (patient-carer).

Table 4 shows the correlation between patients and carer perceptions. There is a statistically significant correlation for emotional functioning for type 2 SMA and the total score for type 3 SMA (Figure 2). In both cases, the correlation is a direct one (i.e., a positive correlation coefficient).

**Table 4:** PedsQL™ 4.0 – association between patient and carer perception (n=33).

PedsQL™ 4.0Domain	Type 1 SMA (n=11)	Type 2 SMA (n=12)	Type 3 SMA (n=10)
Physical functioning	r=0.52 (p=0.100)	r=0.40 (p=0.196)	r=0.60 (p=0.066)
Emotional functioning	r=0.26 (p=0.432)	**r=0.61 (p=0.037)**	r=0.43 (p=0.216)
Social functioning	r=0.31 (p=0.361)	r=0.53 (p=0.074)	r=0.04 (p=0.912)
School functioning	r=0.16 (p=0.656)	r=0.28 (p=0.410)	r=0.62 (p=0.055)
Psychosocial	r=0.11 (p=0.743)	r=0.46 (p=0.132)	r=0.46 (p=0.179)
Total score	r=0.05 (p=0.894)	r=0.27 (p=0.389)	**r=0.72 (p=0.019)**

Results are expressed as Spearman's correlation coefficient and p-value. Bold indicates statistically significant p-values. SMA: spinal muscular atrophy.

## DISCUSSION

The results of this study indicate that there is a difference in perceived QoL between SMA patients and their carers, with the former having a better perception than the latter. Patients with type 2 SMA consider their physical and emotional functioning to be worse than those of patients with other types of SMA. For carers, however, the more severe the type of SMA, the worse the perceived QoL in terms of physical and social functioning. There is a correlation between patient and carer perceptions on emotional functioning for type 2 SMA and between patient and carer total score for type 3 SMA.

This is the first Brazilian study to characterize a large number of SMA patients (235) from all five regions of Brazil. The population sample came from a variety of socioeconomic classes, was distributed equally between the sexes, and had different access to health care. The majority of patients in the sample presented with type 1 SMA (54.5%), followed by type 2 (31.5%), unlike samples in Spain, China, and Thailand, for which the mean percentage when the samples for the three countries were considered together was 60% for type 2 SMA patients.^20-22^

In addition to characterizing SMA patients, the present study considered carers and found that in 46% of the cases, the mother was the main carer. This finding corroborates the systematic review by Brandt et al. and reflects the reality faced by most families with children and adolescents with rare and/or life-limiting diseases.^23^ The fact that it falls upon the mother alone to care for the patient may be related to the low family income observed in this population sample, in which 33% of families live on up to one minimum monthly wage and 37% depend exclusively on the SUS for health care. In another study, lower SMA patient family income was associated with lower QoL.^24^ This correlation was not assessed in the present study, but we can infer that lower family income is associated with worse carer perception.

Although the number of participants attending school regularly was not known, we could observe that all those aged 15–17 years had learned to read and write, and 37.5% of those aged 18 years or older had completed a higher education course. In the population sample from Thailand (n=42), however, approximately 30% of the school-age children did not attend school because of their disability.^21^

In the present study, the number of patients not taking specific medication increased with decreasing disease severity (15% with type 1 SMA, 43% with type 2 SMA, 62% with type 3 SMA, and 100% with type 4 SMA). At the time of writing, only Spinraza (nusinerseen) is provided by the SUS (2020); furthermore, this medication is provided exclusively for patients with types 1 and 2 SMA up to 12 years of age who are not on permanent mechanical ventilation. Duan et al. analyzed the QoL of SMA patients in two groups (with and without medication) and found that patients in the former group had higher HRQoL scores and that their condition improved over time. This finding was observed for all clinical types, including patients who were using mechanical ventilation and a feeding tube.^20^ As medication for SMA in Brazil is only made available to this very specific group of patients, other patients are excluded and thus prevented from achieving favorable clinical outcomes.^25,26^

In Brazil, the physiotherapist is responsible for physical rehabilitation and for handling mechanical ventilators. In the patients in the present study, the assistance provided by the physiotherapist, particularly with respiratory problems, decreased with the decreasing severity of SMA. However, according to a study that analyzed the relationship between multidisciplinary assistance and QoL, even patients with less severe SMA should be assisted by multidisciplinary teams.^27^

In the present study, the carers’ perception agrees with the findings of other studies in which QoL worsened with increasing severity of SMA, except for emotional functioning, for which the inverse was true.^9,20,21,27^ According to the patients’ perception, however, only school functioning decreased with increasing disease severity. Type 2 SMA patients’ perception of QoL in terms of emotional and physical functioning was worse than that of other patients, but their perception of their QoL in terms of social functioning was better. This may reflect the care given to type 1 patients, who receive more attention and achieve better results because they are given specific medication.

Unlike in other studies, both patients and carers considered patients with more severe SMA to have the best QoL in terms of emotional functioning, and for type 2 patients, there was a correlation between patient and carer perception.

In the Thai study, which also compared SMA patient and carer perceptions of QoL, carer scores were lower than patient scores in all the domains. In the present study, in contrast, carer scores were lower in most but not all of the domains. The better QoL perceived by patients than by their carers may be related to the fact that they can adapt to their condition during the course of their lives and take a less serious view of their limitations than their carers.^21^

It is worth remembering that the exact number of patients diagnosed with SMA in Brazil is still unknown. Some limitations of the study should be mentioned. Because it was a remote study, many patients were not involved, and many QoL questionnaires were not answered. This method was chosen due to the restrictions of the COVID-19 pandemic and because it is aimed at patients from all over Brazil.

As there are no reference centers in all Brazilian states, a future form of optimization would be to carry out the study in person in different hospitals in Brazil, thus allowing the collection of a greater number of responses.

Although there is no cure for SMA, assessment of QoL is one way of understanding the reality faced by SMA patients and their carers, and scientific evidence related to QoL still remains low. In the Brazilian population sample studied here, the lower QoL perceived by type 2 SMA patients is noteworthy. This finding should be considered in clinical management and when implementing specific strategies such as greater attention from a multidisciplinary team (especially as far as psychological assistance is concerned), greater availability of medication, and the provision of financial assistance.

Further studies related to QoL should focus on the care provided to patients and their families, early diagnosis, clinical treatment, and multidisciplinary interventions are necessary to better understand the impact on the lives of patients and their caregivers, as well as the epidemiology of the pathology.

This is the first Brazilian study with a significant number of patients diagnosed with SMA. The useful information on SMA patients from the whole of the country in this study provides a broad view of the disorder in Brazil, which can guide clinical treatment and the preparation of public health policies.

## Data Availability

The database that originated the article is available with the corresponding author. CAAE: 39003720.4.0000.0102
